# Effects of diode laser application on inflammation and mpo in periodontal tissues in a rat model

**DOI:** 10.1590/1678-7757-2017-0266

**Published:** 2018-07-06

**Authors:** Mustafa Özay USLU, Abubekir ELTAS, İsmail MARAKOĞLU, Serkan DÜNDAR, Kazım ŞAHIN, İbrahim Hanifi ÖZERCAN

**Affiliations:** 1 Inonu University, Faculty of Dentistry, Department of Periodontology, Malatya, Turkey; 2 Selcuk University, Faculty of Dentistry, Department of Periodontology, Konya, Turkey; 3 Firat University, Faculty of Dentistry, Department of Periodontology, Elazığ, Turkey; 4 Firat University, Veterinary Medicine, Department of Animal Nutrition, Elazığ, Turkey; 5 Firat University, Faculty of Medicine, Department of Pathology, Elazığ, Turkey

**Keywords:** Experimental periodontitis, Inflammation and innate immunity, Laser, Non-surgical periodontal therapy

## Abstract

**Objective:**

In this study, we aimed to histologically and immunologically evaluate the effect of diode laser treatment when applied adjunctive to scaling and root planing (SRP) in an experimental periodontitis model.

**Materials and methods:**

We used Wistar-Albino rats (n=60) with average weight of 230 g. Experimental periodontitis was induced by ligature at the right and left first mandibular molar teeth in all rats. After 11 days, the ligature was removed and rats were divided into two groups. The control group (n=30) received only SRP treatment, while the laser group (n=30) received a diode laser (GaAlAs, 810 nm, 1 W, 10 J, 20 s) treatment adjunctive to SRP. Ten rats in each group were sacrificed after 7, 15, and 30 days. Histopathological examination was performed in the left mandible of rats. Myeloperoxidase (MPO) was evaluated by western blot in the gingival specimens from the right mandible.

**Results:**

MPO levels in the laser group were statistically significantly lower compared with the control group (p≤0.05). There was no statistically significance at any time between MPO levels in the control group (p>0.05). MPO levels in the laser group at the 7^th^ day were statistically significantly higher compared to the 15^th^ (p≤0.05) and the 30^th^ day (p≤0.05). Inflammatory cell infiltration decreased over time in both groups and was statistically significantly lower in the laser group than in the control group at all times (p≤0.01).

**Conclusions:**

Within the limits of this study, we suggest that diode laser application is an adjunctive treatment because it reduced inflammation and MPO when applied in addition to SRP. On the other hand, more studies are needed for the assessment of the effects of diode laser application to periodontal tissues.

## Introduction

Periodontal disease is a chronic inflammatory disorder which develops along with the direct effect caused by the pathogenic bacteria and products in the microbial dental plaque or with the indirect effect through the immune response it poses on the host tissue, resulting in periodontal tissue loss and alveolar bone loss^1^. Apart from several cytokines that play important roles on tissue repair and destruction, inflammatory mediators and proteolytic enzymes as well as inhibitors, the balance between reactive oxygen species (ROS) and the antioxidant defence system also dominates the periodontal destruction^2^.

On the one hand, the cells that are activated and that play a role on the body defence against periodontal diseases can harm microorganisms; on the other hand, free radicals manifest and damage the cells in periodontal tissues as well as the inter-cellular structures. In particular, several ROSs released by polymorphonuclear leukocytes (PMNLs) in the inflammation area cause lipid peroxidation, depolymerization of extra-cellular matrix components and pro-inflammatory cytokines by oxidizing the enzymes, such as anti-proteases, as well as DNA damage. This causes oxidative damage to alveolar bone and periodontal ligaments. Therefore, ROS plays a major role in the pathogenesis of periodontitis^3^.

Myeloperoxidase (MPO) is an enzyme mostly expressed in the azurophilic granules of PMNLs. MPO, which is released extracellularly into phagosome during neutrophil activation and phagocytosis, forms ROS, and it is an important indicator in determining the neutrophil infiltration in the tissues, oxidative stress and tissue damage^4^. Leppilahti, et al.^5^ (2014) found that gingival crevicular fluid MMP-8 and MPO levels in the patients with chronic periodontitis were significantly higher than those with gingivitis and healthy individuals. Wei, et al.^6^ (2004), on the other hand, found that MPO levels in the group with periodontitis were significantly high in compliance with clinical periodontal parameters, and they also reported that ROS played a role on periodontal tissue destruction.

The main target of periodontal treatment is to cease the inflammatory process by reducing the microorganisms through surgical and non-surgical treatments. Nowadays, authors think that the application of laser practices in addition to the non-surgical periodontal treatment could be effective in controlling sub-gingival microorganisms because of their antibacterial efficiency^7^.

Nowadays diode lasers are thought to increase the connective tissue attachment by de-epithelizing the pocket epithelium besides the elimination of the bacteria in the periodontal pockets^8^ ^,^ ^9^. Thus, it was emphasized that the application of diode laser could be used in addition to SRP.

Despite the fact that the use of diode laser in periodontal treatment is on the rise, the results regarding this subject are still contradictory, and further studies need to be conducted^10^ ^-^ ^14^. In particular, the studies in the literature relative to this subject are seen to have been conducted on humans. However, the information is contradictory because of the difficulty in standardizing the oral hygiene and individual differences in humans.

Therefore, in this study, we aimed to evaluate the effects of diode laser treatment on the inflammatory cell infiltration and MPO level in the periodontium when applied adjunctive to SRP in an experimental periodontitis rat model.

## Materials and methods

### Animals

For this study, an approval was received from the Ethics Committee of Laboratory Animals of The Faculty of Medicine in Inonu University. All studies conducted on rats were implemented in The Research and Production Center for Laboratory Animals, Inonu University.

Sixty Wistar-Albino rats with an average weight of 230 g were used during the study period. They were randomly placed in the cages within the same center in the way that there would be five animals in each cage; they were kept at an air-conditioned room with a constant temperature of 20-22ºC. Rats were fed with standard rat feed and water.

### Experimental design

**Figure 1:**
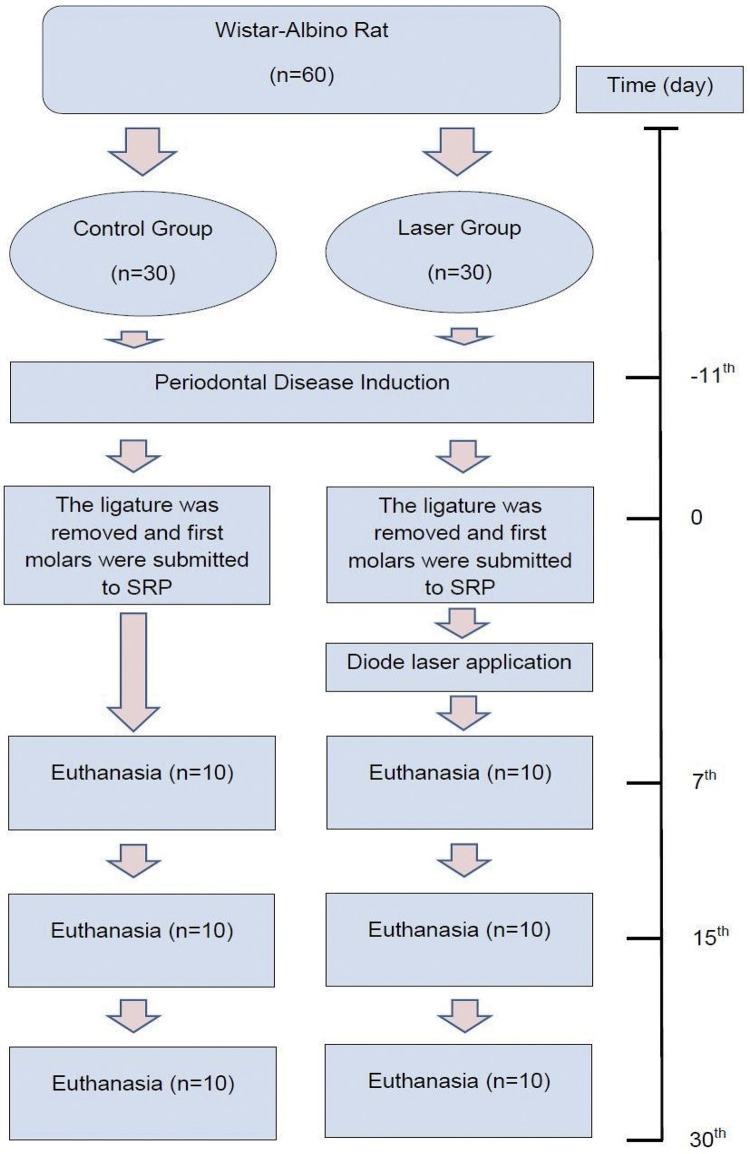
The experimental study model

Figure 1 shows the experimental study model. The minimum sample size of animals was determined by power analysis for sample size calculation based on the data with similar study design given by Bottura, et al.^15^ (2011). As *per* the result of the power analysis performed by using the Power and Sample Size program, the estimated average for the bone loss parameter was determined as 0,4; the standard deviation as 0,2; the power when received was identified as 0,80, whereas the number of samplings determined for α:0,05 was identified as n:5 at miminum for each time period. For each time period, 10 animals were kept, considering the possibility of having drop outs. Sixty rats in total were divided into two groups by being randomly selected using a computer-generated table, in the way that there would be 30 animals in each group. The first animal was the first choice, followed by animals 2 and 3 respectively for better standardization.

Hence, the control group (n=30) received only SRP treatment, WHEREAS The laser group (n=30) was administered diode laser treatment adjunctive to SRP.

### Experimental periodontitis protocol

**Figure 2:**
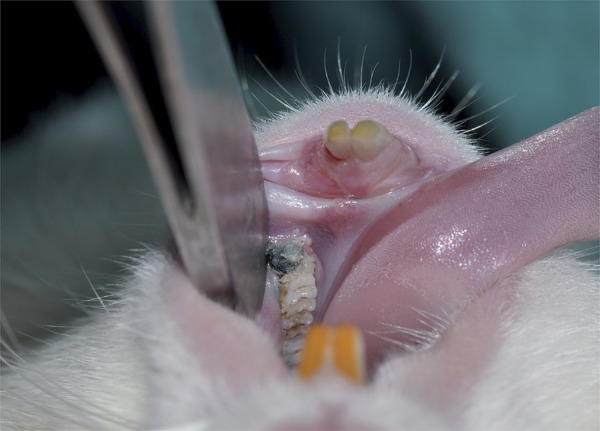
Plaque accumulation and induction of periodontal disease on ligatured first molar tooth at 11th day

Ketasol 30 mg/kg (Richter Pharma AG., Weiz, Austria) and Xylazin Bio 5 mg/kg (Bioveta PLC., Czech Republic) were applied intraperitoneally to all animals. After applying anaesthesia, the measurement of the periodontal pocket depth was performed with conventional calibrated Michigan O probe with Williams markings (Hu-Friedy Co. Inc., Chicago, IL, USA). We evaluated rats regarding periodontal health using probing depth, bleeding on probing, gingival index and plaque index (Sillness&Loe). We passed 3-0 silk suture material (Doğsan, TR) through the first and second mandibular molar teeth in both sides. Ligatures were placed around the first molar teeth in a subgingival region and were then firmly tied to the vestibule. Eleven days after the initiation induction of periodontal disease (Figure 2), the ligature was removed from all animals under anaesthesia and periodontal examination was performed to corroborate the presence of periodontal pockets. The rats that had periodontitis were randomly divided into two groups. One group received only SRP and the other received diode laser treatment adjunctive to SRP at the same day.

### SRP treatment

**Figure 3:**
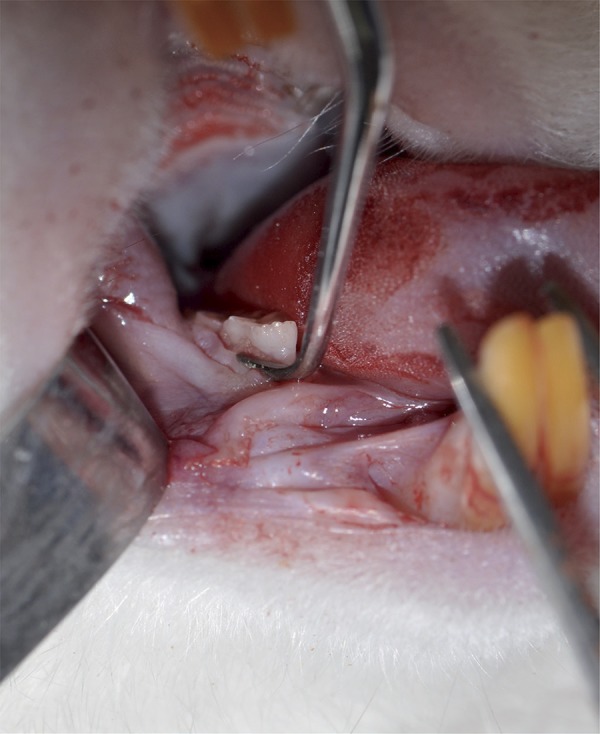
First molars were submitted to SRP with manual n. 1/2 micro mini five Gracey curettes through 10 distal-mesial traction movements in both buccal and lingual aspects

**Figure 4:**
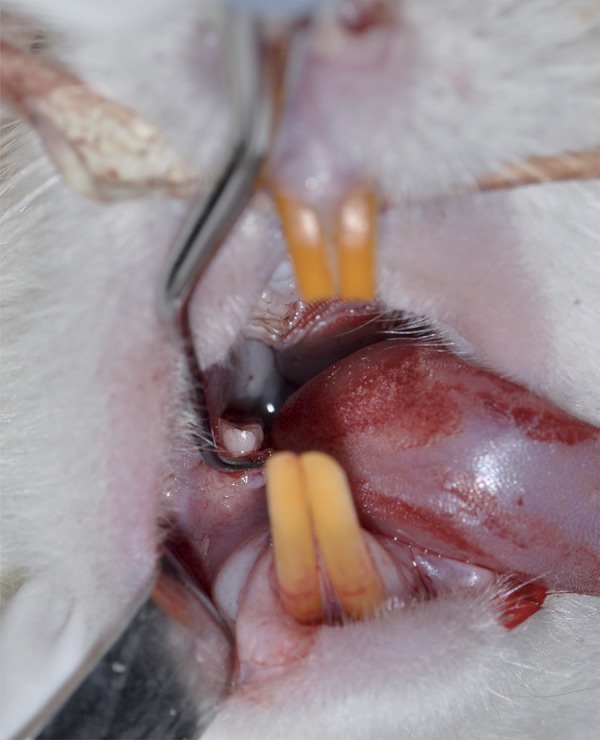
Interproximal areas were scaled with the same curettes through cervico-occlusal traction movements

The rats were anesthetized with ketamine (30 mg/kg) and xylazine (5 mg/kg) by intraperitoneal injection. SRP was applied to first molars with manual n. 1/2 micro mini five Gracey curettes (Hu-Friedy Co. Inc., Chicago, IL, USA). The curettes moved through 10 distal to mesial traction in buccal and lingual sides (Figures 3, 4). The scaling was applied to the furcation and interproximal areas with the same curettes through cervico-occlusal traction movement^16^. The entire SRP protocol was done by the same experienced operator.

### Laser treatment

**Figure 5:**
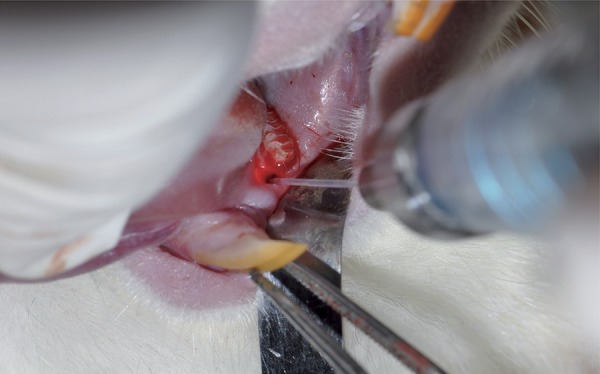
The laser was applied to three equidistant points at each buccal and lingual aspect of the mandibular first molar in contact with the tissue

Similarly to the SRP procedure, rats were anesthetized with ketamine (30 mg/kg) and xylazine (5 mg/kg) by intraperitoneal injection. Gallium-Aluminum-Arsenide (Gigaa Cheese GaAlAs Diode Laser, Wuhan, China) laser application was performed using a wavelength of 810 nm. The laser was applied by optical fiber of 400 µm (spot size of 0.04 cm in diameter and an area of 0.00126 cm^2^) to three equidistant points at both buccal and lingual sides of the first molar in contact mode (power of 1 W, 20 Hz, energy of 10 J/cm^2^, T-on_ms:500, T-off_ms:500, 20 s *per* teeth on continuous mode). In addition to SRP, contact diode laser was applied to the rats in laser group (Figure 5), and hence, the interventions for both of the experimental groups were completed.

### Experimental periods

Ten animals from each group were sacrificed at 7, 15, and 30 days after the periodontal disease treatment under deep anaesthesia. The first molar teeth of the right lower jaw of the sacrificed rats were used for immunological evaluation, while the first molar teeth of their left lower jaw were used for histopathological evaluation.

### Laboratory procedures

#### MPO analysis

Gingival tissues around the right mandibular first molar tooth were collected, pooled together and homogenized. The MPO levels in the tissues were evaluated by using the Western Blot method. Prior to the blotting process, the proteins in the studied samples were made to migrate over the polyacrylamide gel on an electrical platform. The total protein amount contained in the homogenates of the samples was determined according to the Lowry method^17^. The electrophoreses of protein samples were examined through sodium dodecyl sulfate polyacrylamide gel electrophoresis prepared in the way indicated by Laemmli, et al.^18^(1970). MPO rabbit polyclonal antibodies (Santa Cruz Biotechnology, ABD) were used as primary antibodies, which were prepared and used at the ratio of 1:1000 within the buffer including 0.05% viscous liquid (Tween^®^ 20, Sigma-Aldrich). The nitrocellulose membranes along with the primary antibodies were left for incubation at 4°C all night long. To eliminate the uncombined agents, the nitrocellulose membranes were washed with buffer solution five times and for 5 min in the succeeding stage. After the washing process, the nitrocellulose membranes along with the goat anti-rabbit immunoglobulin, which was prepared at the ratio of 1:1000 within the buffer including 0.05% Tween^®^ 20 and was conjugated with peroxidase, were left for incubation at 37°C for 90 min. In the following stage, the nitrocellulose membranes were washed with buffer solution five times and for 5 min. For monitorizing the bands we used 3,3’-Diaminobenzidine tetrahydrochloride (DAB) solution prepared within 1 M Tris (pH: 7,4) buffer at the rate of 0.03-0.05%. As the result of the reaction with DAB, the bands over the nitocellulose membranes became visible after a short while. As the result of a 5-to-10-min-reaction period, the nitrocellulose membranes were washed thoroughly after the bands stained with DAB were clearly seen. After drying them up, the relative densities of the bands were taken to be analyzed the through Image Analyses System program (Image J, National Institute of Health, Bethesda, USA).

#### Histological analyses

**Figure 6:**
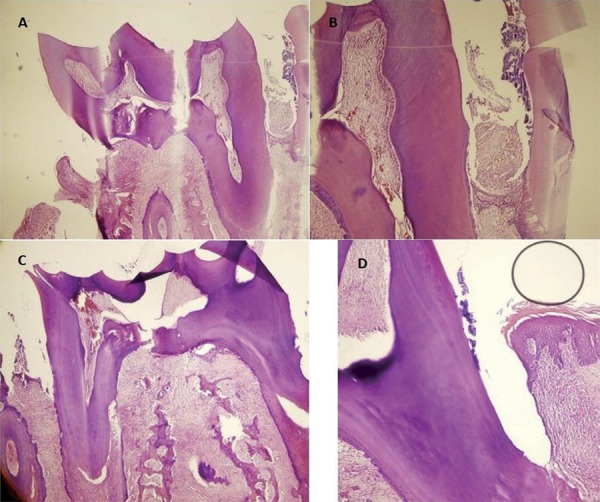
Photomicrograph illustrating the areas of bone loss in the interdental region of the mandibular left first molar with induced periodontal disease in the different groups (control and laser): (A) Control group, scaling and root planing (SRP) treatment for 7 days (H&E, original magnification 40×); (B) Control group (H&E, original magnification 100×); (C) Laser group, administered diode laser adjunctive to SRP treatment for 7 days (H&E, original magnification 40×); (D) Laser group (H&E, original magnification 100×)

The left mandibular teeth, gum and bone tissues to be used in histological examinations were taken into 10%-neutral formaldehyde, and we made their chemical identification. Afterwards, the tissues were taken into a 10%-formic acid solution for 72 hfor the decalcification of samples. After the tissues were made convenient through the cassetting process for the histological examination, we performed dehydratation and transparency processes; then, the tissues were embedded in paraffin blocks. Starting from the initial section where the tooth sections started to be seen longitudinally on the mesio-distal axis, serial paraffin sections were taken via the rotary microtome and were then evaluated. The sections with 4-µm thickness that were taken from all the rat jaws via the microtome (Finesse ME+, Thermo Fisher Scientific, UK) were stained with hematoxylin-eosin (H&E) for the histopathological evaluation of the tissues and for demonstrating and evaluating the collagen fibres as well as the gum tissue (Figures 6 [Fig f07]-8). The tooth and gum tissues pertaining to the groups were evaluated regarding epithelial height, density of collagen fibres and infiltration of the inflammation cell. The following criteria were applied in the evaluation of the findings of inflammation:

**Figure 7 f07:**
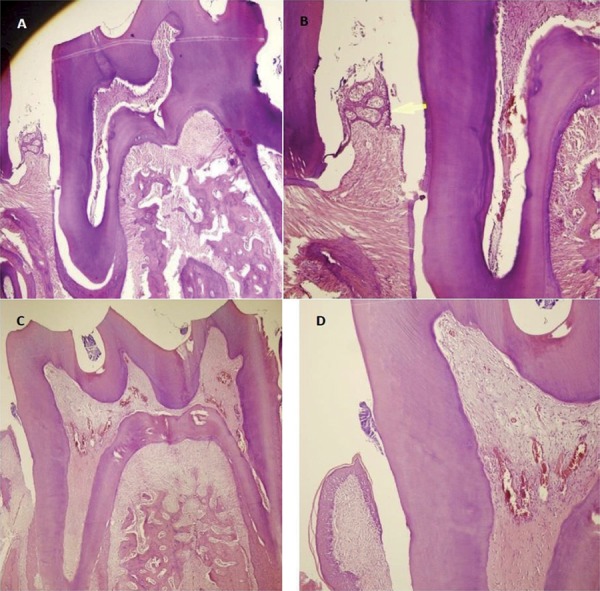
Photomicrograph illustrating the areas of bone loss in the interdental region of the mandibular left first molar with induced periodontal disease in the different groups (control and laser): (A) Control group, SRP treatment for 15 days (H&E, original magnification 40×); (B) Control group (H&E, original magnification 100×); (C) Laser group, administered diode laser adjunctive to SRP treatment for 15 days (H&E, original magnification 40×); (D) Laser group (H&E, original magnification 100×)

**Figure 8:**
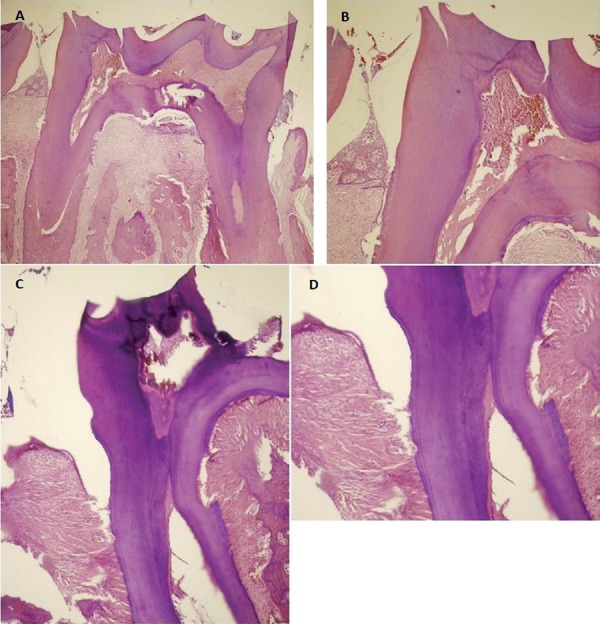
Photomicrograph illustrating the areas of bone loss in the interdental region of the mandibular left first molar with induced periodontal disease in the different groups (control and laser): (A) Control group, SRP treatment for 30 days (H&E, original magnification 40×); (B) Control group (H&E, original magnification 100×); (C) Laser group, administered diode laser adjunctive to SRP treatment for 30 days (H&E, original magnification 40×); (D) Laser group (H&E, original magnification 100×)

Inflammatory cell scoring was performed according to the presence of the cells so as to histopathologically evaluate the inflammatory cell infiltration (ICI)^19^ ^,^ ^20^.

Score 0: The absence of cells; Score 1: The presence of cells on a minimum level; Score 2: The presence of cells on a moderate level; Score 3: The presence of cells on a high level.

As for light microscopy evaluations, the images of the cells, for the inflammatory cell density, were taken with the help of a DP71 camera with an object-glass lens of 40× and 100× magnification that was connected to an Olympus BX51 light microscope (Olympus Corp., Japan) to evaluation. As for the histometric analyses, on the other hand, we used the Olympus software (Olympus DP Controller 3.1.1.267, Japan).

A blinded trained examiner (MT) performed the histological analyses. The values for each selected section were calculated three times with an one-week period between them to reduce data variations. The mean values were calculated and statistically compared.

## Statistical analysis of the data

For the statistical analyses of the findings obtained from the study, we used a statistical software (IBM SPSS Statistics v. 22, USA). The compliance of the data with the normal distribution was evaluated through the Kolmogorov-Smirnov test. During intra-group comparisons, we used Kruskal-Wallis and Mann-Whitney U tests. During the inter-group comparisons, however, we used the Mann-Whitney U test. The significance was evaluated at the level of p≤0.05.

## Results

The MPO levels of the laser group at T1, T2, and T3 time periods were found to be statistically lower on a significant level when compared with the control group (p≤0.05).

**Figure 9:**
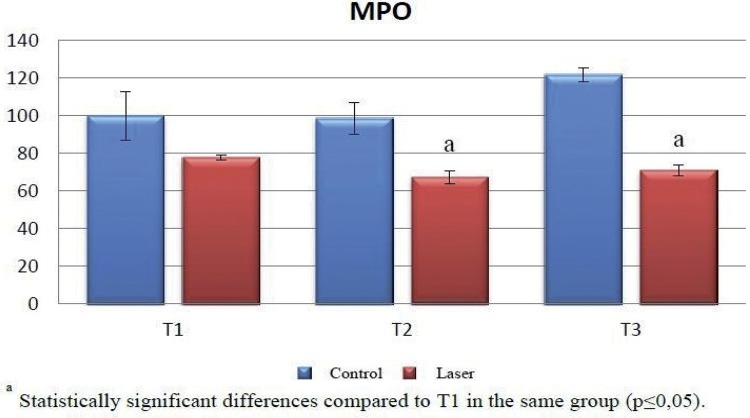
MPO levels in control and laser group. Significant difference among the experimental periods (T1, T2, and T3) in the same group

There was no significant difference between the MPO levels in the control group at T1, T2, and T3 time periods (p>0.05). In the laser group, however, there was a statistically significant difference between MPO levels at T1, T2, and T3 time periods. The MPO level at T1 was statistically higher on a significant level than the MPO levels at T2 (p≤0.05) and T3 (p≤0.05). There was no statistically significant difference between the MPO levels at T2 and T3 (p>0.05) (Table 1) (Figure 1 and 9).

**Table 1:** Mean and standart deviation of myeloperoxidase levels in each group and periods

	Control (n=10)	Laser (n=10)	
MPO	M ± SD	M ± SD	1p
**T1**	100 ± 12.74	77.85 ± 1.51	0.050*
**T2**	98.52 ± 8.27	67.22 ± 3.18	0.050*
**T3**	121.78 ± 3.73	70.89 ± 2.73	0.050*
**2p**	**61**	0.049*	
T1-T2 3p	513	0.050*	
T1-T3 3p	68	0.050*	
T2-T3 3p	75	0.275	

1 Mann-Whitney U test; 2 Kruskal-Wallis test; 3 Mann-Whitney U Test

* p≤0.05

MPO = myeloperoxidase

T1 = 7^th^ day; T2 = 15^th^ day; T3 = 30^th^ day

### Histopathological findings

The ICI levels pertaining to the laser group at the T1, T2, and T3 time periods were found to be statistically lower on a significant level when compared with the control group (p≤0.01).

**Figure 10:**
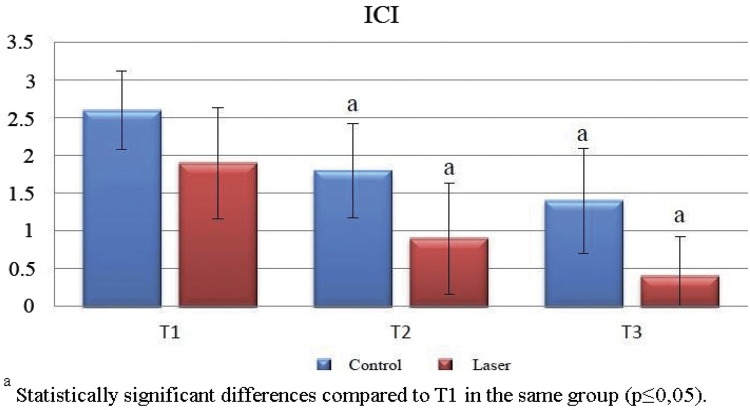
ICI levels in control and laser group. Significant difference among the experimental periods (T1, T2, and T3) in the same group

There was a statistically significant difference between ICI levels at T1, T2 and T3 time periods in both groups. The ICI level at T1 was statistically higher on a significant level than the ICI levels at T2 and T3. There was no statistically significant difference between the ICI levels at T2 and T3 (Table 2) (Figure 10 [Fig f11]).

**Table 2:** Mean and standart deviation of inflammatory cell infiltration levels in each group and periods

	Control (n=10)	Laser (n=10)	
ICI	M ± SD	M ± SD	1p
**T1**	2.6 ± 0.52	1.9 ± 0.74	0.032*
**T2**	1.8 ± 0.63	0.9 ± 0.74	0.013*
**T3**	1.4 ± 0.7	0.4 ± 0.52	0.005**
**2p**	0.002**	0.001**	
T1-T2 3p	0.011*	0.012*	
T1-T3 3p	0.001**	0.001**	
T2-T3 3p	0.235	0.112	

1 Mann-Whitney U test; 2 Kruskal-Wallis test; 3 Mann-Whitney U Test

* p≤0.05

** p≤0.01

MPO = myeloperoxidase

T1 = 7^th^ day; T2 = 15^th^ day; T3 = 30^th^ day

**Figure 11 f11:**
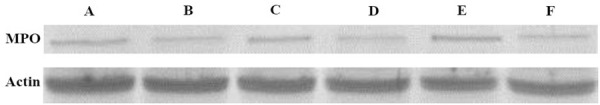
Western blotting bands of MPO and actin. A: Control at 7th day, B: Laser at 7th day, C: Control at 15th day, D: Laser at 15th day, E: Control at 30th day, F: Laser at 30th day

## Discussion

This study evaluated the effects of diode laser application in addition to SRP on inflammation and MPO, which was demonstrated by the local findings of oxidative stress, typical of periodontal disease. At the end of the study, we found that the diode laser had reduced the inflammation and oxidative stress in the periodontal tissues.

In the clinical studies conducted regarding periodontal diseases, there may be contradictions in results due to individual differences, such as the susceptibility of the individual to the progression of the disease, the level of disease activity and the risk factors, which led the researchers to conduct experimental studies^21^. Animal models provide valuable information in investigating the pathogenesis of periodontal disease as well as treatment methods. Apart from the immunological and microbiological characteristics of rats, the histological characteristics of periodontal collagen fibrils, alveolar bone, cellular-non-cellular cementum, the connective tissue, junctional epithelium, oral gingival epithelium and sulcular epithelium show resemblance to human periodontal tissues. Contrary to those in humans, the epithelium of the gingival sulcus is keratinized in rats^22^. Despite this structural difference, the junctional epithelium creates a passageway for bacterial products, foreign substances or inflammatory exudations^21^ ^,^ ^23^ ^,^ ^24^. Because of these characteristics, we also selected rats as the animal model, as in several experimental studies on periodontitis^25^ ^-^ ^28^.

Normally, periodontitis can be initiated in the rats resistant to periodontal diseases through the use of methods such as bacterial vaccination (bacterin), creating a surgical defect, feeding rats with a diet rich in carbohydrates or receiving ligature around the teeth. Since the experimental periodontitis model created by receiving ligature to molar teeth is the model which is closest to the natural plaque formation, it has been used frequently in several studies^16^ ^,^ ^25^ ^,^ ^26^ ^,^ ^29^ ^-^ ^31^. There are studies suggesting that the destruction rate of the alveolar bone was mostly seen on 7^th^-11^th^ days after the ligature had been connected, and that this rate continued decreasing afterwards^26^ ^,^ ^31^ ^,^ ^32^. In this study, the ligatures were removed after the 11^th^ day after the ligatures had been connected to the lower molar teeth, and following SRP and diode laser practices, the rats were sacrificed and evaluated on the 7^th^, 15^th^, and 30^th^ day. The sacrification period of rats was arranged and performed in the way that it would be in compliance with the literature^25^ ^,^ ^29^.

Several treatment options have been applied so far in the treatment of periodontal diseases in which the bacterial dental plaque is the main etiological factor. The main purpose of these treatments is to achieve a smooth root surface by removing tartar and dental plaque to regain the biological connection.

The SRP process is accepted as the golden standard in the non-surgical periodontal treatment and it has successfully found an area of use up until today. However, SRP fails to be effective alone in the presence of furcation areas, concavities and deep pockets. Thus, supplementary methods have been developed in addition to SRP, or surgical interventions have been applied^33^ ^-^ ^35^.

Because of their properties, diode lasers are typically used as soft-tissue lasers. The real reason why diode lasers are preferred in the treatment of periodontitis is the bactericidal effects posed when they are applied in addition to the conventional periodontal treatment.

In a study conducted on patients with periodontitis, Borrajo, et al.^36^(2004) investigated the clinical efficiency of the diode laser used in addition to SRP. Prior to the treatment and in 6^th^ week after the treatment, clinical parameters such as probing pocket depth (PPD), papillary hemorrhage index, clinical attachment loss (CAL), bleeding on probing (BOP) and gingival recession were evaluated, and it was seen that using the diode laser in addition to SRP had ensured a clinically moderate development when compared with the mechanical treatment alone. In contrast, Zare, et al.^37^ (2014) evaluated the effects of diode laser on gingival inflammation after nonsurgical treatment and even though BOP, gingival level and modified gingival index improved in both SRP and SRP+laser groups by time, the indices were not different between two groups except for BOP, which was lower in laser group. In another study^38^ it was mentioned that compared to SRP alone, multiple adjunctive applications of a 980-nm diode laser with SRP showed PD improvements only in moderate periodontal pockets (4 to 6 mm), which shows the limited effects of diode laser on clinical parameters.

Apart from the studies in the literature that promote the fact that the application of diode laser in periodontal treatment is useful regarding microbial and clinical parameters, there are also researchers putting forward the fact that the diode laser used together with SRP has no additional benefit. Micheli, et al.^39^ investigated the non-surgical periodontal treatment assisted by diode laser on patients with chronic periodontitis from clinical and bacterial aspects. While only SRP was applied to one of the groups, diode laser treatment in addition to SRP was performed on the other group on 1^st^ and 7^th^ days. While CAL and PPD results were better in the control group, plaque index (PI) and BOP proved to be similar in both groups. The plaque samples were evaluated at the outset and six weeks later, and the bacteria, such as *P. gingivalis*, *P. intermedia* and *A. actinomycetemcomitans*, were found to be at similar rates in both groups, and it was claimed that the diode laser application did not provide an additional benefit to the conventional periodontal treatment.

In periodontitis, PMNLs activation occurs under the effect of periodontopathogenic bacteria and their virulence factors, some prostaglandins and cytokines. Apart from its antibacterial effects, the MPO enzyme, released from the azurophilic granules of neutrophils, is, under the effect of oxidative stress, also accepted to be the main source of the activation of the latent form of MMP-8 and MMP-9 responsible for the matrix destruction^40^ ^,^ ^41^. As far as we know, there is no other study that has been conducted on the effects of diode laser on ROS, except for the study conducted by Balasubramaniam, et al.^42^(2014). In our study, we aimed to make an evaluation as to the effect of diode laser on the oxidative stress by examining the MPO level. Balasubramaniam, et al.^42^(2014) evaluated in the short term the effects of the use of diode laser in addition to SRP on clinical parameters such PI, BOP, PPD, and CAL and reactive oxygen metabolites while treating the patients with chronic periodontitis. Reactive oxygen metabolites were seen to have decreased on a significant level both in the group to which SRP was applied and in the group to which additional laser was applied, whereas no significant difference was determined between the two groups. In our study, on the other hand, we seen that the MPO level is lower in the laser group and that its tendency to decrease continues up to T3, while it tends to increase in the control group. These results indicate the fact that the laser has positive effects on the oxidative stress, reducing the inflammation. Separately, neutrophils play a major role both in MPO production and in the ICI process. The fact that the MPO level and ICI proved to be reduced in the laser group suggests that the laser may cause a decrease in neutrophil production.

Neutrophils are the major protectors of host defence against microorganisms. Therefore, the findings of our study promote the fact that the laser could reduce the oxidative stress with its antibacterial activity.

In the treatment of periodontitis, the effect of diode laser application on inflammatory changes was determined by evaluating the change in the bacterial load as well as the clinical parameters in the experimental and clinical studies. It was stated that the application of diode laser in addition to SRP alleviated inflammation by reducing the pathogenic bacteria, such as *A. actinomycetemcomitans*, *P. intermedia,* and *P. gingivalis*, ensuring significant recoveries in periodontal tissues^8^ ^,^ ^9^ ^,^ ^43^. One study demonstrated that diode laser was effective on inflammation in the treatment of ligature-induced periodontitis in rats both when used alone and when used as adjunctive therapy to SRP^44^. In our study, we aimed to describe the local inflammatory changes in periodontitis through ICI. While ICI decreased in both groups as time went by, we determined that this decrease was more pronounced in the laser group.

These findings are in compliance with the study results reporting that the application of diode laser alleviates the inflammation in periodontal tissues^9^ ^,^ ^45^ ^,^ ^46^. In the control group, since there were more leukocytes in the region where inflammation had occurred when compared with the laser group, more MPO production may have been generated by these cells. The fact that the decrease in the MPO level is accordant with the decrease in ICI level is supported by the results of the studies in the literature that report that neutrophils are important source of MPO production^40^ ^,^ ^41^. Separately, besides our findings suggesting that inflammation is alleviated when the ICI level decreases, they are also accordant with the studies in the literature that suggest that diode laser alleviates inflammation with its bactericidal effect and enhances the recovery of tissues^8^ ^,^ ^9^ ^,^ ^44^ ^,^ ^46^.

When we review the studies in the literature regarding the application of diode laser, we can see that there is no standard procedure as to the energy level of the laser as well as its mode and period of application into the periodontal pocket. Moreover, we can consider that studies have been performed rather on single-root teeth; yet, the efficiency of laser could be more prominent on multiple-root teeth. The evidence regarding the role of diode lasers (810-980 nm) as adjuncts to SRP in the treatment of chronic periodontitis was systematically reviewed by Qadri, et al.^47^(2015). In this current review, we see that five clinical studies proposed that SRP plus diode laser application was more effective than SRP. However, in two studies, there was a moderate reduction in periodontal inflammation using SRP plus diode laser and three studies showed no difference between laser group and SRP alone. These studies explain the possible contradiction between the use of many parameters such as the diameter of optic fiber (300 μm and 2 mm), laser wavelengths (810-980 nm), power (0.8-2.5 W), pulse repetition rate (10-60 Hz) and duration of laser exposure (10-100 ms) and the use of them at different ranges of values. The presence of all of these variables hinders a more sensitive analysis to be performed on the results and may also be the reason for the contradiction/conflict between those who promote the fact that diode laser has an additional benefit to the non-surgical periodontal treatment and the researchers who claim that it has no additional contribution to the treatment.

This study has a couple of limitations: only the results of diode laser treatment along with SRP were investigated. There is no group in which periodontitis was generated but no treatment was applied. Moreover, there is no group in which periodontitis was generated and treated only with diode laser application. Thus, the pre- and post-treatment levels of the parameters we studied could not be compared. Additionally, only one dose of diode laser application in ligature-induced periodontitis may not be sufficient to assess the actual effectiveness of the diode laser therapy. Thus, the effectiveness of diode laser therapy applied at more frequent and repeated doses should be investigated.

## Conclusion

The results of this study provide significant evidence suggesting that the application of diode laser along with SRP could make additional contribution to the alleviation of inflammation and oxidative stress in periodontal tissues. The fact that there is no standard procedure as to the mode, energy level or the application period of diode lasers used in periodontal treatment requires further studies to be conducted on this type of laser. We are of the opinion that the clinical effect of the diode laser in periodontal treatment will gain much more importance along with the developments in this subject.
